# The Association Between Personality Traits and eSports Performance

**DOI:** 10.3389/fpsyg.2020.01490

**Published:** 2020-07-10

**Authors:** Piotr Matuszewski, Paweł Dobrowolski, Bogdan Zawadzki

**Affiliations:** ^1^Faculty of Psychology, University of Warsaw, Warsaw, Poland; ^2^Institute of Psychology, Polish Academy of Sciences, Warsaw, Poland

**Keywords:** Big Five, personality traits, eSports, League of Legends, performance

## Abstract

This study focuses on the relationship between personality traits, derived from the Big Five model, and performance in the competitive electronic sports (eSports) video game League of Legends (LoL). Data were gathered from 206 LoL players of various in-game success levels, as measured by their position within the competitive hierarchy (division) of the video game. The NEO-Five Factor Inventory (NEO-FFI) questionnaire was used to measure personality traits within the gathered sample, which was divided into two groups of higher and lower ranked players. The results indicate that players who reach higher divisions in LoL tend to be less extraverted, less agreeable, but more open to experience. This is one of the few reported links between eSports performance and personality traits, in what is still a nascent yet rapidly developing research topic. The results are discussed within the context of available knowledge on personality and traditional competitive sports performance.

## Introduction

According to the Global Games Market Report (Newzoo, 2017), there are some 2.2 billion video game players worldwide. While the popularity of video games as a leisure and entertainment activity has visibly grown to such proportions since the advent of home computing, a more recent branch of video-game-related activity is gaining traction worldwide: electronic sports (eSports). Hamari and Sjöblom (2017), p. 211 define eSports as “a form of sports where the primary aspects of the sport are facilitated by electronic systems; the input of players and teams as well as the output of the eSports system are mediated by human–computer interfaces.”

Similar to traditional sports, video games have a substantial number of genres (types). Their unique mechanics attract different types of people, as shown by the relationship between personality traits and video games preferences (Zammitto, 2010; Borders, 2012; Peever et al., 2012). As in typical sports, eSports can take the form of either individual or team-based competition. Moreover, eSports titles have specific rules and schemes, which are typically acquired through the years of hard practice (Himmelstein et al., 2017). The clearest differentiating factor is of course the lack of physical activity. However, eSports professionals must be well coordinated due to the demands of their interface (mouse and keyboard, gamepads) and must also have a great deal of composure and control over their bodies to avoid interference effects like inaccurate sweeping movements while controlling the mouse (Witkowski, 2012). Despite these similarities, there is currently no knowledge regarding which factors contribute to success in eSports.

Some expectations can be formed based on the literature relating to expert performance in traditional sports, although this comparison is a distant one at best due to the understandable focus of that research on the physical aspects of performance. One potential point of overlap, and the topic of this study, is the relationship between performance and personality traits. Participation (Kirkcaldy, 1985; Kirkcaldy and Furnham, 1991) and performance (Kirkcaldy, 1982; Kerr and Cox, 1991; Egloff and Gruhn, 1996; Khan et al., 2016) in traditional sports have been shown to relate positively with extraversion. One study indicated that only conscientiousness could be considered a valid predictor of traditional sports success (Mirzaei et al., 2013), and two others have shown that higher levels of conscientiousness and lower levels of neuroticism predict athletic performance (Piedmont et al., 1999) and participation in national or international competitions (Allen and Laborde, 2014). Further, two studies have indicated that superior athletes scored higher in conscientiousness and agreeableness, as well as lower in neuroticism (Allen et al., 2011; Steca et al., 2018). Concurrently, individuals with higher levels of neuroticism are more prone to mind wandering than those with lower levels of neuroticism (Robison et al., 2017) and tend to select less adaptive coping strategies (Allen et al., 2011; Kaiseler et al., 2012). Given the fact that emotional stability is generally beneficial for player performance in sports (Raglin, 2001) and for non-sport games like Poker (Laakasuo et al., 2014), it may interlay into the video gaming context. Video game genres within the eSports sphere are both competitive and fast paced; as such, emotional stability may be crucial for reaching an optimal level of performance. Finally, it is also worth noting that researchers have previously shown a relationship between personality traits and participation in either individual or team sports, indicating that team sports participants tend to be more agreeable (Nia and Besharat, 2010) and extraverted (Eagleton et al., 2007).

The purpose of the current study is to explore the relationship between eSports performance and personality traits in order to begin building a base of knowledge about this accelerating social phenomenon. We chose to focus on the highly popular eSports video game League of Legends (LoL). LoL belongs to the leading genre (in terms of players and watchers) in eSports, namely, Multiplayer Online Battle Arena (MOBA). Despite its popularity (Mora-Cantallops and Sicilia, 2018), research regarding LoL and other MOBA games is scarce in the context of personality traits, mainly addressing the issue of problematic usage (Nuyens et al., 2016). Originally released in 2009 by developer Riot Games, LoL gameplay consists of matches that are contested by two teams of five players each. The main goal of each team is to destroy the *Nexus*, which is the final objective in the enemy’s base. Several sub-objectives must be completed along the way, and players strive to complete them while also defending against the opposite team. Being a team game where players have to be cohesive in the pursuit of a common goal (Pereira et al., 2016), LoL is somewhat comparable in function to other team-based sports. Its high level of competitiveness has garnered an ever-increasing number of viewers; the 2011 League of Legends World Championship final attracted some 210,000 viewers (The Escapist, 2011), while in 2018, that number jumped to almost 200 million (ESCharts, 2018).

Although some parallels can be drawn between eSports and traditional sports, forming any predictions about the links between eSports performance and personality traits based on the data available for the latter would be speculative at best. As such, this study provides an exploratory overview of this relationship by measuring the personality traits (as measured by the NEO inventory) of LoL players who vary in their level of in-game performance and achievement. That being said, given the nature of competitive and team-based endeavors, we suspect that lower neuroticism and higher levels of extraversion, conscientiousness, and agreeableness may be predictive of superior performance in LoL.

## Materials and Methods

### Participants

The study sample was comprised of LoL players recruited via Facebook groups dedicated to the LoL community. An invitation to complete an online questionnaire was posted to these groups. The total amount of records consisted of 1,697 participants, out of which 206 (18 female) completed the entire questionnaire and were included in the analyses. Prior to providing data, participants were informed of their rights as voluntary subjects (in accord with the Helsinki Declaration), and informed consent was gathered via confirmation on the questionnaire’s landing page. Sociodemographic data were collected in the first part of the questionnaire, including age, gender, and level of education. The age of the participants varied between 18 and 27 years of age (*M* = 19.99, *SD* = 1.88), and they held the following levels of education: secondary (35%), primary (25.2%), secondary vocational (21.8%), higher education (8.3%), postsecondary (6.3%), and vocational (3.4%). This study was assessed and approved by the local ethics committee.

### Measures

League of Legends performance was operationalized here as position within the ranking ladder. Players could be in one of nine divisions, from best to worst: Challenger, Master, Diamond, Platinum, Gold, Silver, and Bronze. This information was gathered and verified by asking the study participants for their LoL nickname, which is a unique identifier that allows for their accounts to be viewed online. This also allowed us to verify that each participant was a current LoL player, as it provides data on recent gameplay activity.

The following phase of the questionnaire included online version of the NEO-Five Factor Inventory (NEO-FFI). The NEO-FFI questionnaire is a shortened version of the NEO Personality Inventory (NEO-PI), which is a measure of five personality traits derived from the Big Five Personality Factors developed by Costa and McCrae (1989). It consists of 60 items, 12 per each factor. Those factors are neuroticism (α = 0.86), extraversion (α = 0.80), openness (α = 0.60), agreeableness (α = 0.78), and conscientiousness (α = 0.82).

## Results

**FIGURE 1:**
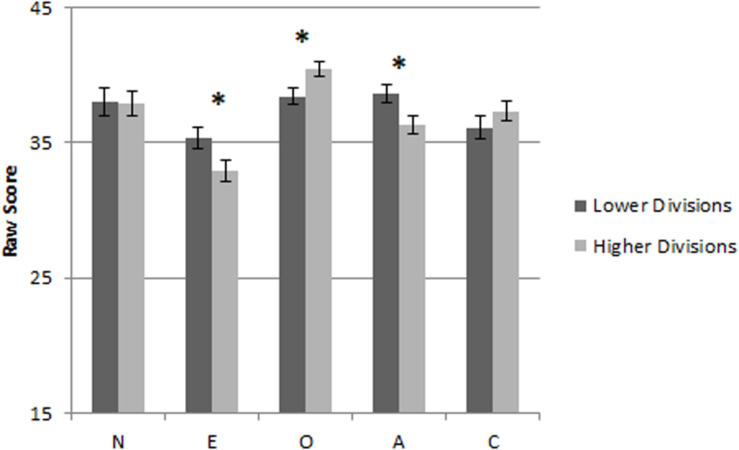
Personality traits raw scores comparison between lower and higher divisions.

For the purposes of statistical analysis, our participants were divided into two groups of “lower” (*n* = 102) and “higher” (*n* = 104) divisions. As we had no respondents from the Challenger division, we created the groups by combining the three lowest (Bronze, Silver, and Gold) and three highest (Platinum, Diamond, and Master) divisions that were represented in our data (Figure 1). The groups were subsequently compared on their results in each of the five personality traits using multivariate ANOVA (MANOVA). Age, gender, and education level were controlled for by adding them to the model as covariates (see Table 1 for details).

**TABLE 1:** MANOVA results for comparison of lower and higher ranked players.

	Lower Rank		Higher Rank					
	*M*	*SD*	*M*	*SD*	*F*	*df*	*p*	η_*p*_^2^
Neuroticism	38.05	10.33	37.92	9.59	0.01	1	0.924	0.000
Extraversion	35.34	7.81	32.91	7.75	5.05	1	0.026	0.025
Openness	38.43	5.92	40.44	5.85	6.23	1	0.013	0.030
Agreeableness	38.65	7.03	36.32	7.19	4.72	1	0.031	0.023
Conscientiousness	36.14	8.28	37.35	7.50	1.43	1	0.234	0.007

*Effect of covariates age [*F*(5, 197) = 2.88, *p* = 0.016, η_*p*_^2^ = 0.068], gender [*F*(5, 197) = 1.41, *p* = 0.222, η_*p*_^2^ = 0.035], and education [*F*(5, 197) = 0.96, *p* = 0.447, η_*p*_^2^ = 0.024].*

There was a significant difference between the lower and higher ranked players on dependent variables of neuroticism, extraversion, openness, agreeableness, and conscientiousness: *F*(5, 197) = 4.24, *p* = 0.001, η*_p_*^2^ = 0.097. Players within the lower division group had significantly higher levels of extraversion [*F*(1, 197) = 5.05, *p* = 0.026, η*_p_*^2^ = 0.025] and agreeableness [*F*(1, 197) = 4.72, *p* = 0.031, η*_p_*^2^ = 0.023] while also having a lower level of openness [*F*(1, 197) = 6.23, *p* = 0.013, η*_p_*^2^ = 0.030]. However, it should be noted that the effect sizes of these results are relatively small.

## Discussion

The main purpose of this study was to examine the link between personality traits and success in LoL. Our results indicate that the traits of extraversion, agreeableness, and openness significantly differ between lower and higher ranking LoL players. Due to the team-based nature of LoL gameplay, one may expect that the traits of extraversion and agreeableness relate positively to performance. This was not the case, with the lower ranked players exhibiting significantly higher scores on both of those traits than the higher ranked players. This result is opposite to that reported in research on team-based sports players (Eagleton et al., 2007; Nia and Besharat, 2010), although lower agreeableness has previously been linked to better sports performance (Khan et al., 2016).

Lower agreeableness and extraversion in the higher division group may be explained by the fact that the ranking is measured by individual performance. It may be possible that players who are highly focused on themselves during the game and do everything to “carry the game” on their own are more successful. Despite the fact that LoL is a team game, teams, and hence coplayers, change with every ranking game. This means that there is a high degree of variance in terms of team performance from match to match, which may mean that a self-centered approach to the game is a good strategy to maximize ranking in the long-term. Openness also differed between our groups, but with higher ranking players scoring higher on the trait than lower ranked players. People with higher levels of openness tend to be more flexible and creative. In the context of LoL, which is a game that is constantly changing over time and hence requires players to adapt, it may be the case that players with lower levels of that trait struggle to adjust to the variable game environment and hence perform worse.

The other trait that we expected to differ in our two groups was neuroticism. It is well established that athletes (Piedmont et al., 1999; Raglin, 2001; Allen et al., 2011; Allen and Laborde, 2014; Khan et al., 2016; Steca et al., 2018) as well as poker players (Laakasuo et al., 2014) exhibit high emotional stability, i.e., they are even tempered and are less susceptible to negative emotions like stress or worry. In our study, the results show no significant relationship between neuroticism and LoL performance. It may be the case that our sample, which consisted of competitive but not professional players, did not experience the same levels of pressure to perform as professional athletes. Since a high level of neuroticism predicts higher stressor intensity and lower stressor control (Kaiseler et al., 2012), emotional stability might be crucial for professional LoL players, but not amateurs. In the case of conscientiousness, as it is an indicator of individuals being hard working, organized, persistent, and achievement oriented (Costa et al., 1995), we might have expected a positive relationship with LoL performance. We did not find such a relationship, and it may again be the difference between true professionals and avid players. It is perhaps not surprising, as video games are primarily a form of entertainment.

It is important to note that this study is not free from limitations. First of all, given the observational and cross-sectional nature of this study, the inference regarding causality between personality traits and eSports performance is limited. Second, because of the nature of the dependent variables, it was necessary to divide the whole study group into two subgroups based on the division indicator. A continuous indicator of performance such as match-making rating would be preferable, as well as a larger sample size (to increase statistical power) and the inclusion of Challenger division players into the analysis. In addition, given the paucity of female respondents in our study, the current results cannot be generalized to players of both genders. Furthermore, in the present study, only one game within one particular genre was examined. It would be of great interest to see if the personality correlates of performance differ between types of games. As game genres are generally defined by unique gameplay mechanics, and these mechanics place unique demands on their players, it is likely that different aspects of personality will be most related to performance across genres. Lastly, as LoL is a team game, further research should examine whether professional players and non-professional, high division players differ. Since LoL professionals are part of five person rosters, they may have different characteristics to be able to constantly cooperate in such teams.

In summary, we were able to demonstrate a link between performance in LoL and the personality traits of extraversion, openness, and agreeableness. Our results only partly overlap with the sports literature (namely on the traits of agreeableness and openness), which suggests that eSports, as a competitive activity, require or attract a unique set of personality traits. However, this comparison should be interpreted with caution, due to the general paucity of data on eSports performance and personality. To conclude, this study provides the first evidence – to the best of our knowledge – of a link between video game performance and personality in general and thus opens a new avenue of research. We hope that future studies will add to this initial body of knowledge to further elucidate the nature of this relationship.

## Data Availability Statement

The datasets generated for this study are available on request to the corresponding author.

## Ethics Statement

The studies involving human participants were reviewed and approved by the Institute of Psychology, Polish Academy of Sciences. The participants provided their written informed consent to participate in this study.

## Author Contributions

PM: idea of the study, participants recruitment, data analysis, and main author of the manuscript. PD: extension of research idea, help in the research and data analysis plan, formal and lingual correction of the manuscript. BZ: help in the research and data analysis plan, formal correction of the manuscript. All authors contributed to the article and approved the submitted version.

## Conflict of Interest

The authors declare that the research was conducted in the absence of any commercial or financial relationships that could be construed as a potential conflict of interest.
